# Supplementary material for: Spodoptera frugiperda (Lepidoptera: Noctuidae) Life Table Comparisons and Gut Microbiome Analysis Reared on Corn Varieties
Source: Insects. 2023 Apr 4;14(4):358. doi: 10.3390/insects14040358 (PMC10146201; doi:10.3390/insects14040358)
Supplement: Supplementary file 1 [file insects-14-00358-s001.zip › insects-2276380-supplementary.pdf]

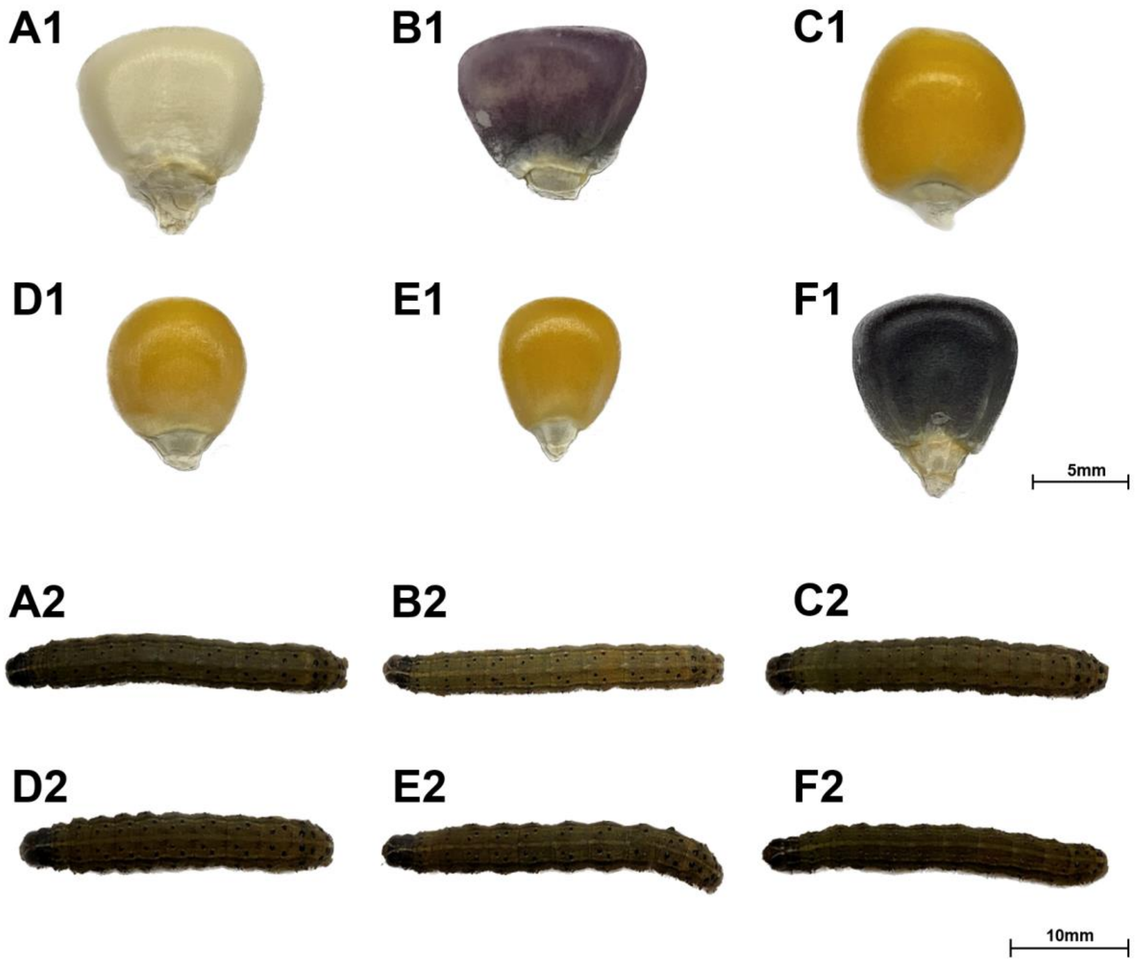

Figure S1: The morphological features of commercialized six maize corn and *Spodoptera frugiperda* larvae that fed on them. (A1) mebaek 2-ho, whitish color (B1) heukjeom 2-ho, blue color (C1) dreamok, yellow color (D1) oryun popcorn, yellow color (E1) oryun 2-ho, yellow color and (F1) meheukchal, purple color. Three (n=3) commercially available waxy corn cultivars (mebaek 2-ho, heukjeom 2-ho, and meheukchal), two (n=2) popcorn cultivars (oryun popcorn, oryun 2-ho), and one (n=1) processing purpose cultivar (dreamok). The six different larvae are depicted as A2-F2. A2 larvae consumes A1 corn-seedlings; similarly, B2, C2, D2, E2 and F2 consume corn varieties: B1, C1, D1, E1 and F1, respectively.

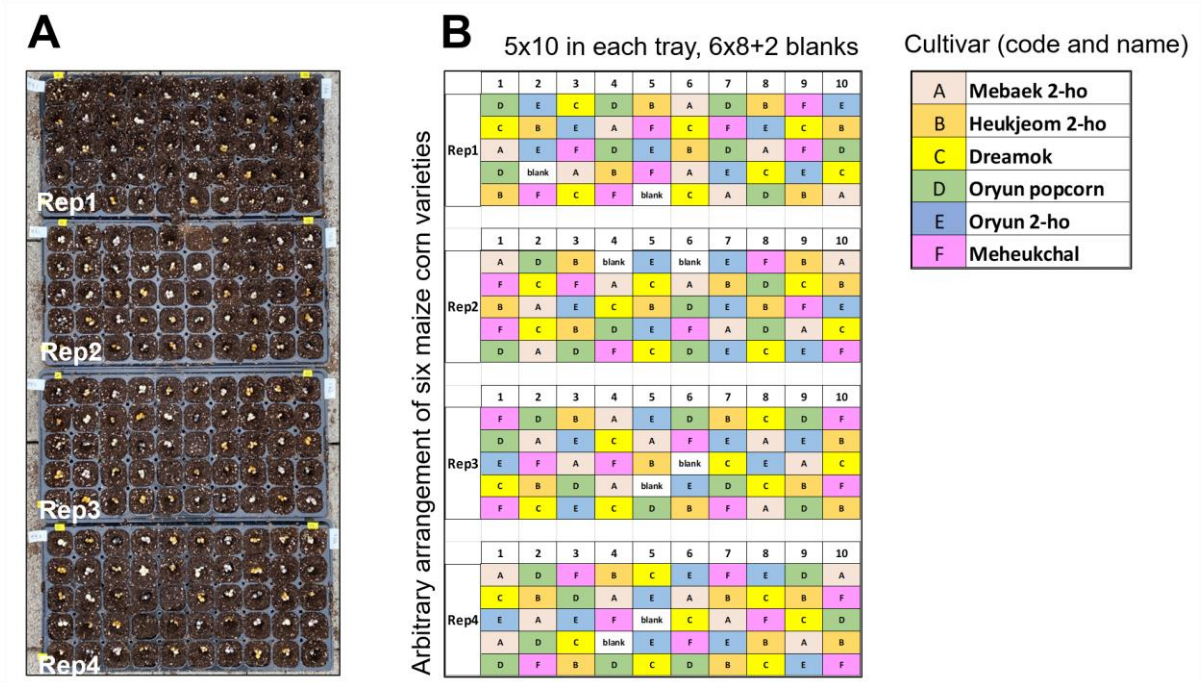

Figure S2: Random selection and arbitrary arrangement of six corn varieties (A) Actual early-stage view of maize seedlings in a plastic tray on commercial bed soil (replication as rep1–4). (B) Six corn variety deployment layers. Random selection was executed over four replications for seven days, sowing eight corn seeds of six corn varieties in 5 × 10 trays (including two blank spots). (2022.06.21 – 2022.06.27);

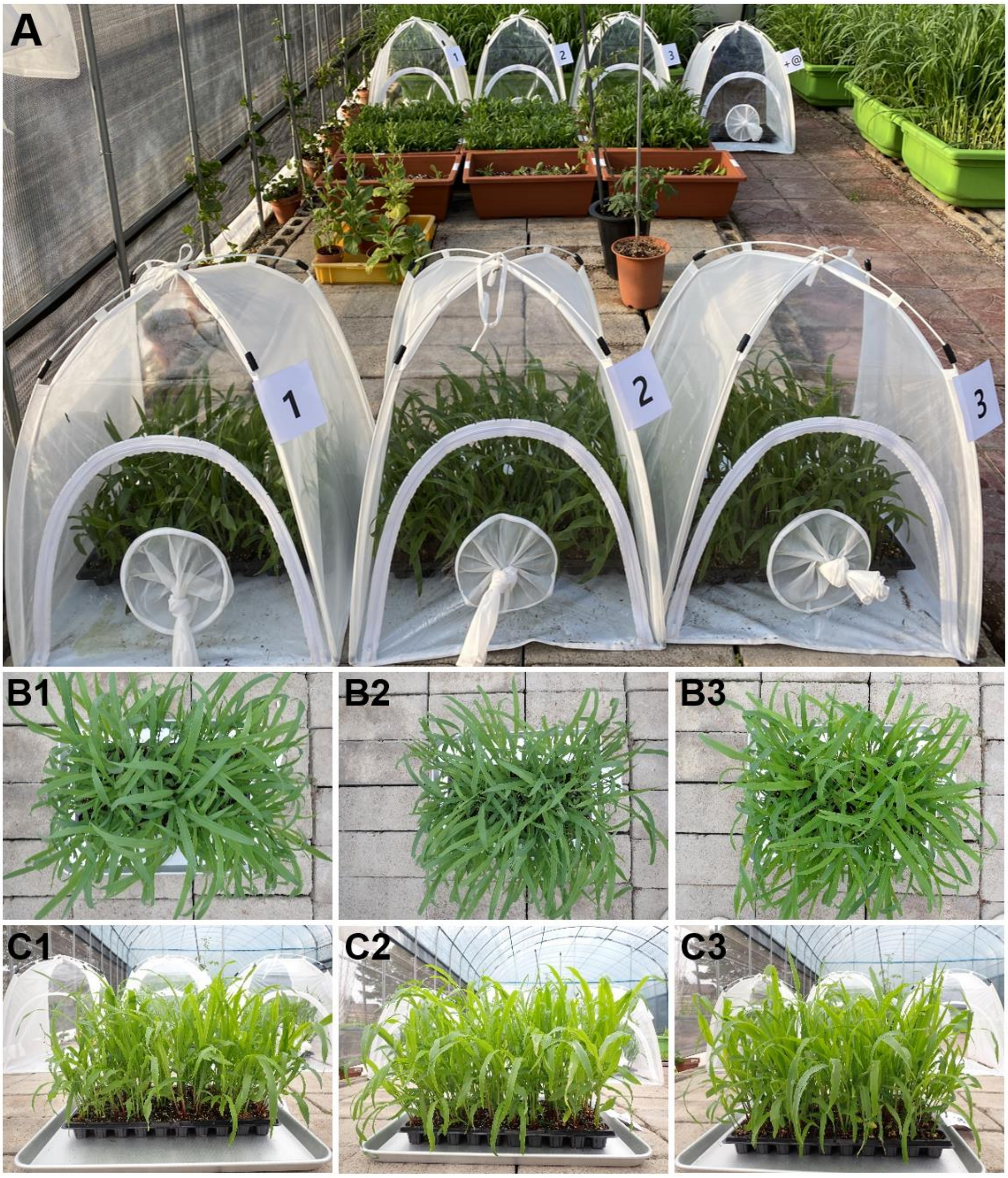

Figure S3: Six commercialized maize corn cultivars were grown in a greenhouse at Chuncheon campus, Kangwon National University, in order to understand the host preferences of *Spodoptera frugiperda*. (A) The experiment was conducted in a BugDorm-2400 Insect Rearing Tent (75 × 75 × 115) mebaek 2-ho, (B) heukjeom 2-ho, (B1-B3) Top view after two weeks (each variety of corn reached a height of approximately 50 cm), (C1-C3) side view after two weeks (each variety of corn reached a height of approximately 50 cm);

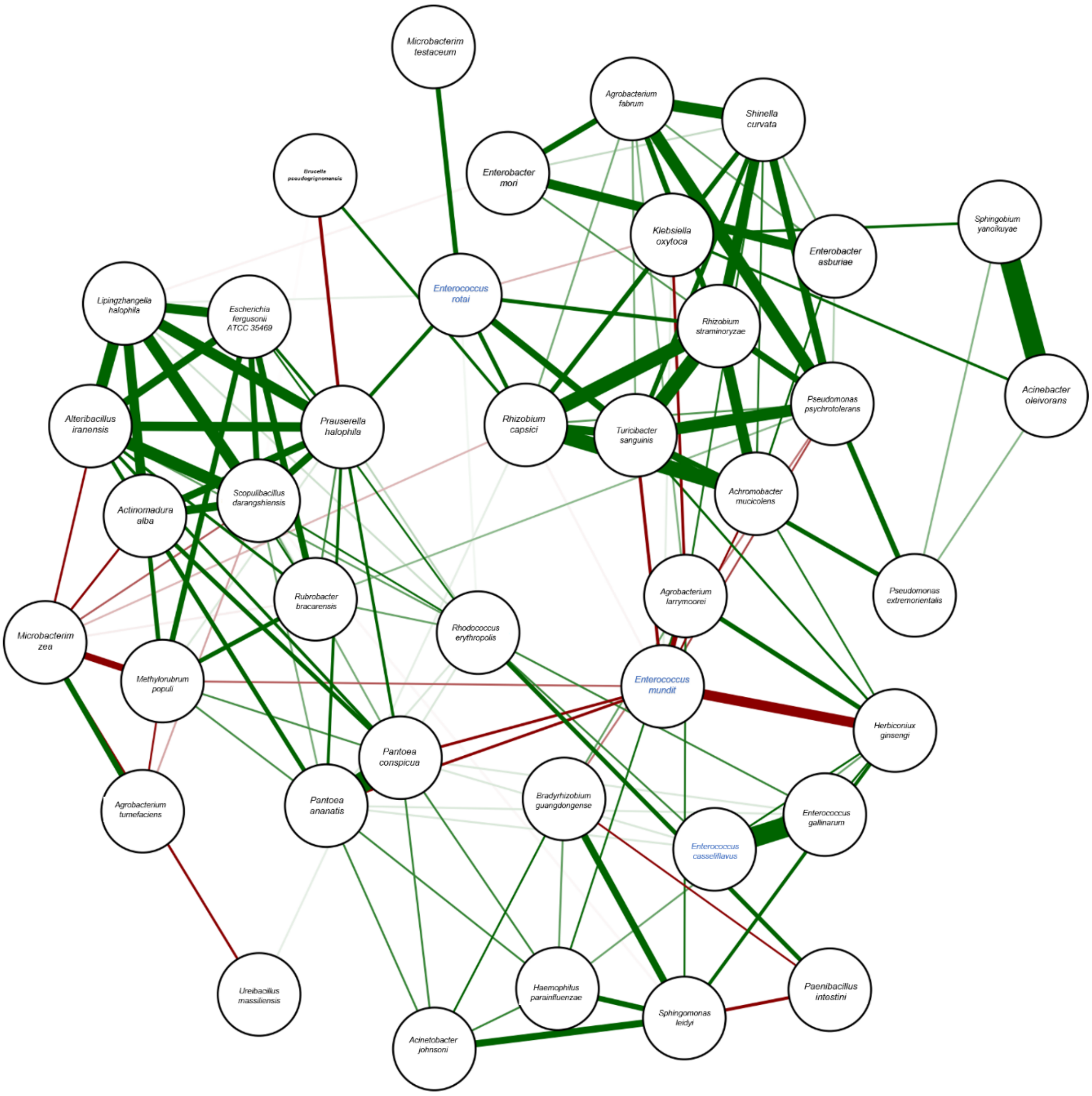

Figure S4: Species richness and network analysis of bacterial species showes a interction to the bacterial community up to top forty species. The most dominant *Enterococcus* spp. are presented in the middle of each circle by blue color and rest of the bacteria are presented by black color. Green line indicates a positive relationship, red line indicates a negative relationship. The arbitrary line thickness shows the relation level of each bacterial population.

Table S1 The information of paired reads, filtered and trimmed reads from 18 insect gut samples

Sample	Input reads (paired)	Filtered reads[1)]	Merged reads[2)]	Non-chimeric reads[3)]	Contaminant removal[4)]
FAW_A1	95,076	26,951	26,863	26,842	20,410
FAW_A2	75,636	26,079	25,724	25,454	19,188
FAW_A3	92,702	29,579	29,241	28,542	23,271
FAW_B1	105,281	33,819	33,662	33,588	30,657
FAW_B2	110,780	31,127	30,880	30,782	27,673
FAW_B3	131,126	46,091	45,828	45,724	43,224
FAW_C1	120,800	43,303	43,188	43,110	40,480
FAW_C2	119,016	26,263	26,032	25,793	23,943
FAW_C3	116,073	29,433	29,292	29,243	26,703
FAW_D1	141,109	48,816	48,535	48,425	43,990
FAW_D2	116,627	31,123	30,882	30,667	26,659
FAW_D3	127,434	36,192	35,900	35,723	31,995
FAW_E1	129,657	44,942	44,878	44,800	43,082
FAW_E2	111,958	35,774	35,568	34,926	31,135
FAW_E3	121,062	44,057	43,929	43,737	42,705
FAW_F1	99,374	33,880	33,829	33,826	31,634
FAW_F2	108,559	37,605	37,509	37,456	36,119
FAW_F3	51,542	15,178	15,130	15,112	13,919

[1)] Calculate the q-value based on the trimmed length set for the input reads, and remove reads that do not meet the criteria.

[2)] Merge the two reads, which must overlap by at least 12 bp to merge.

[3)] Compare ASV(amplicon sequence variant)s to select only non-chimeric reads.

[4)] Remove contaminants (almost human-derived sequences) of 400 bp or less that are significantly smaller than the size of the target region (about 450 bp) and rRNA sequences derived from plastids, including chloroplasts and mitochondria.
